# 2-[(4,6-Dimeth­oxy­pyrimidin-2-yl)­oxy]benzaldehyde

**DOI:** 10.1107/S1600536812000840

**Published:** 2012-01-18

**Authors:** Xue-Jun He, Jun-Song Song, Qin-Qin Huang, De-Cai Wang, Ping-Kai Ou-yang

**Affiliations:** a State Key Laboratory of Materials-Oriented Chemical Engineering, School of Pharmaceutical Sciences, Nanjing University of Technology, Xinmofan Road No. 5 Nanjing, Nanjing 210009, People’s Republic of China.

## Abstract

In the title compound, C_13_H_12_N_2_O_4_, the dihedral angle between the benzene and pyrimidine rings is 55.57 (13)°. The carbonyl group and the two methoxyl groups are approximately coplanar with the benzene ring and pyrimidine ring; the C—C—C—O, C—O—C—N and C—O—C—C torsion angles being −6.1 (5), −4.8 (4) and 179.9 (3)°, respectively. In the crystal, mol­ecules are linked *via* C—H⋯O inter­actions, forming chains propagating along [110].

## Related literature

For the synthesis of the title compound, see: Yang & Lu (2010 ▶).
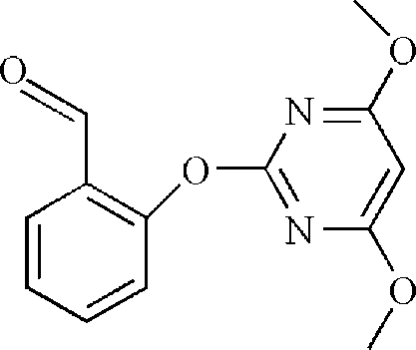


## Experimental

### 

#### Crystal data

C_13_H_12_N_2_O_4_
*M* *_r_* = 260.25
Monoclinic, 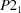

*a* = 3.9920 (8) Å
*b* = 7.3670 (15) Å
*c* = 20.885 (4) Å
β = 94.87 (3)°
*V* = 612.0 (2) Å^3^
*Z* = 2
Mo *K*α radiation
μ = 0.11 mm^−1^
*T* = 293 K
0.30 × 0.20 × 0.10 mm

#### Data collection

Enraf–Nonius CAD-4 diffractometer
Absorption correction: ψ scan (*SADABS*; Sheldrick, 1996 ▶) *T*_min_ = 0.969, *T*_max_ = 0.989
2563 measured reflections
2227 independent reflections
1790 reflections with *I* > 2σ(*I*)
*R*_int_ = 0.024
3 standard reflections every 200 reflections intensity decay: 1%

#### Refinement

*R*[*F* ^2^ > 2σ(*F* ^2^)] = 0.046
*wR*(*F* ^2^) = 0.131
*S* = 1.00
2227 reflections
172 parameters
1 restraint
H-atom parameters constrained
Δρ_max_ = 0.14 e Å^−3^
Δρ_min_ = −0.14 e Å^−3^

### 

Data collection: *CAD-4 EXPRESS* (Enraf–Nonius, 1994 ▶); cell refinement: *CAD-4 EXPRESS*; data reduction: *XCAD4* (Harms & Wocadlo, 1995 ▶); program(s) used to solve structure: *SHELXS97* (Sheldrick, 2008 ▶); program(s) used to refine structure: *SHELXL97* (Sheldrick, 2008 ▶); molecular graphics: *SHELXTL* (Sheldrick, 2008 ▶); software used to prepare material for publication: *SHELXTL*.

## Supplementary Material

Crystal structure: contains datablock(s) I, global. DOI: 10.1107/S1600536812000840/su2359sup1.cif

Structure factors: contains datablock(s) I. DOI: 10.1107/S1600536812000840/su2359Isup2.hkl

Supplementary material file. DOI: 10.1107/S1600536812000840/su2359Isup3.cml

Additional supplementary materials:  crystallographic information; 3D view; checkCIF report

## Figures and Tables

**Table 1:** Hydrogen-bond geometry (Å, °)

*D*—H⋯*A*	*D*—H	H⋯*A*	*D*⋯*A*	*D*—H⋯*A*
C2—H2*B*⋯O1^i^	0.93	2.54	3.400 (4)	154

Symmetry code: (i) 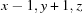.

**Fig. 1.**
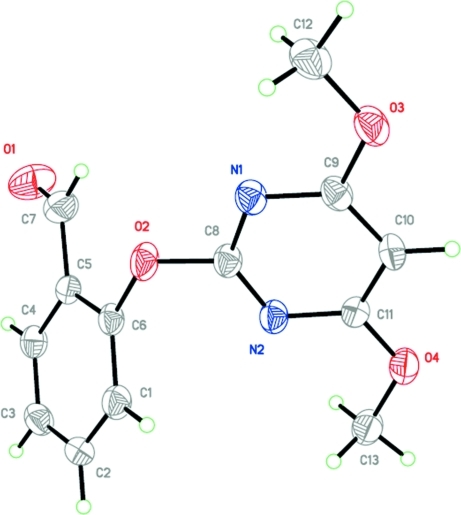
The molecular structure of the title compound, showing the atomic numbering scheme and displacement ellipsoids drawn at the 30% probability level.

## supplementary crystallographic information

### Comment

The title compound is an important organic intermediate for the synthesis of 2-pyrimidine-oxy-*N*-aryl benzyl amine derivatives, which are important compounds for new pesticides. In the process of synthesising one such derivative we obtained crystals of the title compound, and we report herein on its crystal structure.

As illustrated in Fig. 1, the molecular structure of the title compound is not planar, the dihedral angle between the (C1—C6) benzene and the (N1/N2,C8-C10) pyrimidine rings is 55.57 (13) °. The carbonyl group and the two methoxyl groups are approximately coplanar with the benzene ring and pyrimidine ring, as shown by the torsion angles C4—C5—C7—O1 = -6.1 (5) °, C13—O4—C11—N2 = -4.8 (4) ° and C12—O3—C9—C10 = 179.9 (3) °.

In the crystal, there is a C-H···O interaction present (Table 1), that results in the formation of chains that run along direction [110].

### Experimental

The title compound was synthesized according to the published procedure (Yang & Lu, 2010). A solution of 12.2 g salicylaldehyde, 21.8 g of 2,6-dimethoxy-4- (Methylsulfonyl) Pyrimidine, and 41.4 g of K_2_CO_3,_ in 150 ml acetonitrile, was heated to 220 K for 4 h. The solution was then filtered, and the filtrates were concentrated under reduced pressure. Colourless block-like crystals of the title compund, suitable for X-ray diffraction, were obtained by slow evaporation of the solvent [Yield 86%].

### Refinement

The C-bound H atoms were placed in calculated positions and treated as riding atoms: C—H = 0.93 and 0.96 Å for CH and CH_3_ and H atoms, respectively, with U_iso_(H) = k × U_eq_(C), where k = 1.5 for CH_3_ H-atoms and k = 1.2 for all other H-atoms. In the final cycles of refinement, in the absence of significant anomalous scattering effects, 1023 Friedel pairs were merged and Δf " set to zero.

### Crystal data

**Table:**

C_13_H_12_N_2_O_4_	*F*(000) = 272
*M**_r_* = 260.25	*D*_x_ = 1.412 Mg m^−^^3^
Monoclinic, *P*2_1_	Mo *K*α radiation, λ = 0.71073 Å
Hall symbol: P 2yb	Cell parameters from 25 reflections
*a* = 3.9920 (8) Å	θ = 9–13°
*b* = 7.3670 (15) Å	µ = 0.11 mm^−^^1^
*c* = 20.885 (4) Å	*T* = 293 K
β = 94.87 (3)°	Block, colourless
*V* = 612.0 (2) Å^3^	0.30 × 0.20 × 0.10 mm
*Z* = 2	

### Data collection

**Table:**

Enraf–Nonius CAD-4 diffractometer	1790 reflections with *I* > 2σ(*I*)
Radiation source: fine-focus sealed tube	*R*_int_ = 0.024
graphite	θ_max_ = 25.4°, θ_min_ = 2.0°
ω/2θ scans	*h* = 0→4
Absorption correction: ψ scan (*SADABS*; Sheldrick, 1996)	*k* = −8→8
*T*_min_ = 0.969, *T*_max_ = 0.989	*l* = −25→25
2563 measured reflections	3 standard reflections every 200 reflections
2227 independent reflections	intensity decay: 1%

### Refinement

**Table:**

Refinement on *F*^2^	Primary atom site location: structure-invariant direct methods
Least-squares matrix: full	Secondary atom site location: difference Fourier map
*R*[*F*^2^ > 2σ(*F*^2^)] = 0.046	Hydrogen site location: inferred from neighbouring sites
*wR*(*F*^2^) = 0.131	H-atom parameters constrained
*S* = 1.00	*w* = 1/[σ^2^(*F*_o_^2^) + (0.085*P*)^2^] where *P* = (*F*_o_^2^ + 2*F*_c_^2^)/3
2227 reflections	(Δ/σ)_max_ < 0.001
172 parameters	Δρ_max_ = 0.14 e Å^−^^3^
1 restraint	Δρ_min_ = −0.14 e Å^−^^3^

### Special details

**Table:**

Geometry. Bond distances, angles etc. have been calculated using the rounded fractional coordinates. All su's are estimated from the variances of the (full) variance-covariance matrix. The cell esds are taken into account in the estimation of distances, angles and torsion angles
Refinement. Refinement of *F*^2^ against ALL reflections. The weighted *R*-factor *wR* and goodness of fit *S* are based on *F*^2^, conventional *R*-factors *R* are based on *F*, with *F* set to zero for negative *F*^2^. The threshold expression of *F*^2^ > σ(*F*^2^) is used only for calculating *R*-factors(gt) *etc*. and is not relevant to the choice of reflections for refinement. *R*-factors based on *F*^2^ are statistically about twice as large as those based on *F*, and *R*- factors based on ALL data will be even larger.

### Fractional atomic coordinates and isotropic or equivalent isotropic displacement parameters (Å^2^)

**Table:**

	*x*	*y*	*z*	*U*_iso_*/*U*_eq_	
O1	1.4553 (7)	0.5127 (4)	0.39674 (15)	0.0925 (11)	
O2	0.8453 (6)	0.7927 (3)	0.27202 (8)	0.0581 (7)	
O3	0.7500 (5)	0.7425 (3)	0.05537 (8)	0.0591 (8)	
O4	1.2359 (6)	1.2581 (3)	0.15175 (9)	0.0598 (8)	
N1	0.7955 (6)	0.7671 (3)	0.16578 (10)	0.0457 (8)	
N2	1.0511 (6)	1.0293 (3)	0.21505 (10)	0.0460 (7)	
C1	0.8229 (7)	1.0480 (4)	0.34509 (13)	0.0503 (9)	
C2	0.9064 (7)	1.1207 (4)	0.40473 (14)	0.0515 (10)	
C3	1.1033 (7)	1.0252 (4)	0.44989 (14)	0.0518 (10)	
C4	1.2202 (7)	0.8574 (4)	0.43577 (13)	0.0460 (9)	
C5	1.1421 (7)	0.7798 (4)	0.37569 (12)	0.0413 (8)	
C6	0.9419 (7)	0.8792 (4)	0.33036 (12)	0.0411 (8)	
C7	1.2647 (9)	0.5998 (4)	0.36179 (17)	0.0638 (11)	
C8	0.9023 (7)	0.8697 (4)	0.21501 (12)	0.0432 (9)	
C9	0.8461 (7)	0.8376 (4)	0.10859 (12)	0.0444 (9)	
C10	0.9930 (7)	1.0035 (4)	0.10102 (13)	0.0501 (10)	
C11	1.0929 (7)	1.0950 (4)	0.15650 (13)	0.0433 (8)	
C12	0.5970 (8)	0.5691 (5)	0.06309 (14)	0.0629 (11)	
C13	1.3137 (9)	1.3609 (4)	0.20875 (15)	0.0583 (11)	
H1B	0.68640	1.11240	0.31480	0.0600*	
H2B	0.82900	1.23560	0.41460	0.0620*	
H3A	1.15740	1.07510	0.49040	0.0620*	
H4A	1.35410	0.79390	0.46680	0.0550*	
H7A	1.18890	0.54810	0.32260	0.0760*	
H10A	1.02300	1.05120	0.06070	0.0600*	
H12A	0.54180	0.51540	0.02160	0.0940*	
H12B	0.39590	0.58390	0.08470	0.0940*	
H12C	0.75070	0.49160	0.08810	0.0940*	
H13A	1.41400	1.47410	0.19800	0.0880*	
H13B	1.46830	1.29400	0.23750	0.0880*	
H13C	1.11130	1.38390	0.22920	0.0880*	

### Atomic displacement parameters (Å^2^)

**Table:**

	*U*^11^	*U*^22^	*U*^33^	*U*^12^	*U*^13^	*U*^23^
O1	0.1009 (19)	0.0614 (16)	0.112 (2)	0.0237 (15)	−0.0101 (16)	0.0028 (15)
O2	0.0881 (15)	0.0545 (12)	0.0307 (10)	−0.0212 (11)	−0.0003 (9)	0.0054 (8)
O3	0.0734 (15)	0.0684 (14)	0.0350 (11)	−0.0096 (12)	0.0019 (10)	−0.0057 (9)
O4	0.0798 (15)	0.0533 (12)	0.0477 (12)	−0.0107 (12)	0.0134 (11)	0.0053 (9)
N1	0.0502 (13)	0.0510 (14)	0.0349 (12)	−0.0028 (11)	−0.0023 (9)	0.0010 (9)
N2	0.0527 (13)	0.0485 (13)	0.0366 (12)	−0.0027 (12)	0.0026 (10)	0.0050 (10)
C1	0.0556 (17)	0.0529 (17)	0.0426 (15)	0.0039 (14)	0.0052 (13)	0.0098 (13)
C2	0.0619 (18)	0.0447 (16)	0.0505 (17)	0.0076 (14)	0.0196 (14)	0.0002 (12)
C3	0.0563 (16)	0.0596 (19)	0.0405 (15)	−0.0086 (16)	0.0102 (12)	−0.0091 (13)
C4	0.0465 (15)	0.0540 (17)	0.0369 (14)	−0.0036 (13)	−0.0002 (11)	0.0059 (12)
C5	0.0481 (15)	0.0390 (14)	0.0375 (14)	−0.0038 (12)	0.0075 (11)	0.0036 (11)
C6	0.0477 (14)	0.0454 (14)	0.0299 (13)	−0.0074 (13)	0.0022 (11)	0.0029 (11)
C7	0.080 (2)	0.0497 (18)	0.0615 (19)	0.0067 (18)	0.0051 (16)	0.0043 (15)
C8	0.0482 (15)	0.0474 (17)	0.0333 (14)	−0.0010 (14)	−0.0007 (11)	0.0052 (11)
C9	0.0455 (14)	0.0550 (17)	0.0320 (14)	0.0048 (13)	−0.0014 (11)	−0.0005 (12)
C10	0.0630 (18)	0.0565 (18)	0.0317 (14)	0.0012 (15)	0.0092 (13)	0.0071 (12)
C11	0.0485 (15)	0.0397 (14)	0.0426 (15)	0.0008 (13)	0.0085 (11)	0.0046 (11)
C12	0.066 (2)	0.074 (2)	0.0476 (17)	−0.0141 (17)	−0.0017 (15)	−0.0155 (16)
C13	0.072 (2)	0.0521 (18)	0.0505 (17)	−0.0099 (17)	0.0037 (15)	0.0043 (13)

### Geometric parameters (Å, °)

**Table:**

O1—C7	1.196 (5)	C5—C6	1.394 (4)
O2—C6	1.400 (3)	C5—C7	1.451 (4)
O2—C8	1.355 (3)	C9—C10	1.371 (4)
O3—C9	1.342 (3)	C10—C11	1.370 (4)
O3—C12	1.431 (4)	C1—H1B	0.9300
O4—C11	1.338 (4)	C2—H2B	0.9300
O4—C13	1.423 (4)	C3—H3A	0.9300
N1—C8	1.318 (3)	C4—H4A	0.9300
N1—C9	1.334 (3)	C7—H7A	0.9300
N2—C8	1.317 (4)	C10—H10A	0.9300
N2—C11	1.339 (3)	C12—H12A	0.9600
C1—C2	1.371 (4)	C12—H12B	0.9600
C1—C6	1.375 (4)	C12—H12C	0.9600
C2—C3	1.370 (4)	C13—H13A	0.9600
C3—C4	1.362 (4)	C13—H13B	0.9600
C4—C5	1.390 (4)	C13—H13C	0.9600
			
C6—O2—C8	121.3 (2)	N2—C11—C10	123.0 (3)
C9—O3—C12	117.9 (2)	C2—C1—H1B	120.00
C11—O4—C13	118.7 (2)	C6—C1—H1B	120.00
C8—N1—C9	114.3 (2)	C1—C2—H2B	120.00
C8—N2—C11	114.4 (2)	C3—C2—H2B	120.00
C2—C1—C6	119.6 (3)	C2—C3—H3A	120.00
C1—C2—C3	120.4 (3)	C4—C3—H3A	120.00
C2—C3—C4	120.3 (3)	C3—C4—H4A	120.00
C3—C4—C5	120.9 (3)	C5—C4—H4A	120.00
C4—C5—C6	118.0 (3)	O1—C7—H7A	117.00
C4—C5—C7	120.2 (3)	C5—C7—H7A	117.00
C6—C5—C7	121.9 (3)	C9—C10—H10A	122.00
O2—C6—C1	122.1 (2)	C11—C10—H10A	122.00
O2—C6—C5	116.9 (3)	O3—C12—H12A	109.00
C1—C6—C5	120.8 (2)	O3—C12—H12B	109.00
O1—C7—C5	125.2 (3)	O3—C12—H12C	109.00
O2—C8—N1	112.2 (2)	H12A—C12—H12B	109.00
O2—C8—N2	118.8 (2)	H12A—C12—H12C	109.00
N1—C8—N2	129.0 (2)	H12B—C12—H12C	109.00
O3—C9—N1	119.0 (3)	O4—C13—H13A	109.00
O3—C9—C10	117.7 (2)	O4—C13—H13B	109.00
N1—C9—C10	123.3 (2)	O4—C13—H13C	110.00
C9—C10—C11	116.0 (3)	H13A—C13—H13B	109.00
O4—C11—N2	118.7 (2)	H13A—C13—H13C	110.00
O4—C11—C10	118.3 (2)	H13B—C13—H13C	109.00
			
C8—O2—C6—C5	−126.4 (3)	C2—C1—C6—O2	175.3 (3)
C6—O2—C8—N1	179.6 (2)	C6—C1—C2—C3	−0.9 (4)
C6—O2—C8—N2	−0.1 (4)	C1—C2—C3—C4	0.5 (4)
C8—O2—C6—C1	59.2 (4)	C2—C3—C4—C5	−0.2 (4)
C12—O3—C9—C10	179.9 (3)	C3—C4—C5—C6	0.3 (4)
C12—O3—C9—N1	0.0 (4)	C3—C4—C5—C7	−179.1 (3)
C13—O4—C11—C10	174.6 (3)	C4—C5—C6—O2	−175.2 (3)
C13—O4—C11—N2	−4.8 (4)	C6—C5—C7—O1	174.6 (3)
C9—N1—C8—N2	−0.4 (4)	C7—C5—C6—C1	178.6 (3)
C8—N1—C9—O3	179.2 (2)	C4—C5—C7—O1	−6.1 (5)
C8—N1—C9—C10	−0.7 (4)	C4—C5—C6—C1	−0.7 (4)
C9—N1—C8—O2	179.9 (2)	C7—C5—C6—O2	4.1 (4)
C11—N2—C8—N1	1.0 (4)	O3—C9—C10—C11	−178.9 (3)
C8—N2—C11—C10	−0.7 (4)	N1—C9—C10—C11	1.0 (4)
C11—N2—C8—O2	−179.3 (3)	C9—C10—C11—N2	−0.2 (4)
C8—N2—C11—O4	178.7 (3)	C9—C10—C11—O4	−179.6 (3)
C2—C1—C6—C5	1.0 (4)		

### Hydrogen-bond geometry (Å, °)

**Table:**

*D*—H···*A*	*D*—H	H···*A*	*D*···*A*	*D*—H···*A*
C2—H2B···O1^i^	0.93	2.54	3.400 (4)	154

Symmetry codes: (i) *x*−1, *y*+1, *z*.
